# Tissue-Resident Lymphocytes Across Innate and Adaptive Lineages

**DOI:** 10.3389/fimmu.2018.02104

**Published:** 2018-09-21

**Authors:** Chun Chou, Ming O. Li

**Affiliations:** Immunology Program, Memorial Sloan Kettering Cancer Center, New York, NY, United States

**Keywords:** tissue resident, innate lymphocyte, innate-like T cells, conventional T cells, cancer, infection

## Abstract

Lymphocytes are an integral component of the immune system. Classically, all lymphocytes were thought to perpetually recirculate between secondary lymphoid organs and only traffic to non-lymphoid tissues upon activation. In recent years, a diverse family of non-circulating lymphocytes have been identified. These include innate lymphocytes, innate-like T cells and a subset of conventional T cells. Spanning the innate-adaptive spectrum, these tissue-resident lymphocytes carry out specialized functions and cross-talk with other immune cell types to maintain tissue integrity and homeostasis both at the steady state and during pathological conditions. In this review, we provide an overview of the heterogeneous tissue-resident lymphocyte populations, discuss their development, and highlight their functions both in the context of microbial infection and cancer.

## Introduction

A fundamental role of the immune system is to maintain host integrity. For metazoan species, an effective immune response must address invading threats in a rapid and specific manner such that the afflicted tissues remain uncompromised and continue to carry out their vital functions for the host. The innate immune system provides the first line of defense through the recognition of stereotypic motifs associated with a broad spectrum of pathogens (1–3). In contrast, the adaptive immune system, equipped with antigen receptors of near-limitless diversity, exerts its effector functions in an antigen specific manner (3). This expanded population of antigen-specific adaptive lymphocytes in turn forms the basis of immunological memory, bestowing the hosts with long-lasting immunity against previously encountered pathogens (3).

For mammalian species, the adaptive immune response is initiated in secondary lymphoid structures by antigen presenting cells (APCs). Upon activation by danger-associated signals, APCs migrate from the site of insult to draining lymph nodes, carrying with them components of the menacing agents. There they present these captured antigens to naïve T lymphocytes, which in turn triggers the successive rounds of cell division by T lymphocytes and initiates their differentiation into effector and memory subsets. Whereas, effector T cells home back to the primary sites of insult, mediate clearance of pathogen and undergo population contraction, memory T cells persist after the resolution of infection and are poised to mount recall responses. Under this classical view, the secondary lymphoid tissues are the integral component of the adaptive immune system, for the constant migration of adaptive lymphocytes within such a network maximizes their chance of antigen encounter (4). Teleologically, this circulatory behavior of naïve adaptive lymphocytes is a necessary consequence of their anticipatory antigen receptor repertoire (5). The antigen receptor genes of adaptive lymphocytes are assembled through random somatic recombination without prior knowledge of their cognate antigen. This anticipatory nature of the adaptive antigen receptor repertoire underlies its tremendous diversity, but greatly limits the frequency of lymphocytes with a given specificity. As such, a given naïve T cell clone cannot be present in all tissues at once. By necessity, they patrol strategically placed lymph nodes, which collect information on the statuses of their associated tissues, to efficiently survey the antigen landscape of the whole organism.

Our understanding of lymphocyte responses has broadened significantly in the past decade by the successive discovery of many non-circulating lymphocyte populations. These lymphocytes predominantly reside in non-lymphoid tissues in stark contrast to naïve adaptive lymphocytes, which constantly recirculate between secondary lymphoid organs. In fact, it is now well-appreciated that many, if not all, non-lymphoid organs harbor a sizable population of tissue-resident lymphocytes. These include tissue-resident memory T (T_RM_) cells; unconventional T cells such as invariant natural killer T (iNKT) cells, intraepithelial lymphocytes (IEL), and γδ T cells; and a diverse family of innate lymphocytes. This property of tissue residency spans across the innate-adaptive spectrum and may be essential for the tissue-specific functions of its respectively resident lymphocyte populations. In this review, we introduce the defining features of tissue-resident lymphocytes, provide an overview of their characteristic features, summarize recent findings on their ontogeny, and discuss their functions in the context of cancer.

## Defining tissue-resident lymphocytes

The defining feature of tissue-resident lymphocytes is their distinct migration pattern. In contrast to naïve adaptive lymphocytes which frequently travel between secondary lymphoid organs, tissue-resident lymphocytes constitutively reside in non-lymphoid tissues and generally do not re-circulate through blood (6, 7). This blood-tissue disequilibrium can be conveniently approximated by intravascular staining (8–10). Intravenous administration of fluorescently-conjugated antibody labels vasculature-associated cell populations in a short period of time. Unlabeled cells are thus presumed to reside in the tissue parenchyma and are unlikely to re-circulate. The tissue resident property is most formally demonstrated by parabiosis experiments in which the circulatory systems of two animals are surgically joined, allowing for free exchange of their cell populations (11). Over time, half of the re-circulating lymphocyte compartment in one animal will be derived from its parabiont (6, 11). In contrast, the non-circulating compartment remains dominated by endogenous lymphocyte populations with little to no input from the parabiont (6, 11). This restricted migratory pattern of tissue-resident lymphocytes is often associated with their lack of lymphoid tissue homing chemokine receptors and elevated expressions of several adhesion molecules (7, 12). The sphingosine-1-phosphate receptor (S1PR1) and the chemokine receptor CCR7, whose ligands, S1P, and CCL19/21 are abundantly found in the blood and secondary lymphoid organs, respectively, facilitate re-circulation of lymphocytes and are downregulated as part of the tissue residency program (13–15). On the contrary, CD69, which antagonizes S1PR1 signaling, is reciprocally upregulated (16, 17). In addition, increased expression of integrin molecules, such as CD49a (encoded by *Itga1*) and CD103 (encoded by *Itgae*), whose ligands are collagen and E-cadherin, respectively, promotes interaction with tissue constituents, further reinforcing retention of lymphocytes (18, 19). Whereas, the downregulation of CCR7 and S1PR1 seems to be universal for tissue-resident lymphocytes, the usage of integrin molecules is more diverse. CD103 is specifically found on lymphocytes associated with epithelial tissues, such as the small intestine epithelium and ductal epithelium in glandular organs (20–23). CD49a and CD69 also have their own tissue-restricted expression patterns (18, 24–26). These observations highlight the substantial heterogeneity within the tissue-resident lymphocyte compartment. Thus, defining tissue-resident populations solely based on phenotypic markers may not reliably identify all cells. Instead, parabiosis experiments remain the gold standard to properly define tissue residency.

## Overview of tissue-resident lymphocyte populations

So far, tissue-resident populations have been identified for all known types of lymphocyte across the innate-adaptive spectrum (6), strongly suggesting that the acquisition of the tissue residency program represents a state of differentiation rather than commitment to a distinct lineage. Resident lymphocyte populations are hypothesized to sense in their home organs tissue disturbances stemming from infection, stress and other deviations from the norm. In turn, they initiate the necessary immune responses to restore homeostasis. Below we briefly describe the characteristic features of various tissue-resident lymphocyte populations and their functions in maintaining tissue integrity.

### Innate lymphocytes

Innate lymphocytes are characterized by their lack of functionally re-arranged antigen receptors. This population includes the prototypic member, natural killer (NK) cells, and the emerging family of innate lymphoid cells (ILCs) (27, 28). Under steady-state conditions, NK cells are recirculating while ILCs are not (6). Emerging evidence suggest that ILCs can be further parsed based on their cytotoxic potential into two subsets: helper ILCs, which are IL-7R-expressing cytokine producers, and killer ILCs, which express cytotoxic molecules but have little to no IL-7R expression (28). Helper ILCs are enriched at mucosal sites and include ILC1, ILC2, and ILC3, each of which produces signature cytokines not unlike their helper T cell subset counterparts (27). The killer ILCs, on the other hand, are mostly found in the liver and epithelium of glandular tissues, such as the salivary, prostate, and mammary glands, and can mediate direct cytolysis of target host cells through granzyme secretion or Fas ligand engagement (23, 29–31).

The exact function of tissue-resident type 1 innate lymphocytes remains contentious. Because of their striking resemblance to NK cells at the phenotypic level, studies aiming to test NK cell functions by depleting NK marker-expressing populations through antibodies or diphtheria toxin system may have inadvertently eliminated type 1 ILCs as well. Hence it is difficult to pinpoint which population mediates the observed phenotypes. This caveat has only been recognized recently but nevertheless precipitated the development of new genetic tools to selectively target either populations. For instance, a recent study utilized animals deficient for the transcription factor *Zfp683*, or *Hobit*, to specifically reduce the number of liver ILCs, leaving the NK compartment intact (32). In these animals, control of early viral replication in the liver was impaired, supporting the idea that resident type 1 ILCs function as first line defenders.

Type 2 ILCs are the most homogenous among the innate lymphocytes and produce signature cytokines of the type 2 response, such as IL-5, IL-13, and amphiregulin, in a transcription factor *Gata3*-, *Bcl11b*, and *Rora*-dependent manner (33–35). ILC2s control normal immune responses through cross-talk between stroma and other immune cell types. For instance, during helminth infection, intestinal tuft cell-derived IL-25 activates ILC2s to secrete IL-13, which feedbacks on the epithelium to promote tuft cell differentiation (36). The alarmin IL-33 produced upon tissue injury also stimulates IL-5 production by ILC2s, which in turn recruits eosinophils and enhance their innate effector functions (37). This pathway can be antagonized by a secretory product of the helminth *H. polygyrus*, HpARI, which prevents the release of IL-33 by tethering it to necrotic cells (38), further demonstrating the evolutionary benefit of ILC2-dependent responses.

Group 3 ILCs are highly complex and can be roughly unified by their dependency on the transcription *Rorc* for development and function (39). Upon activation by IL-23, a subset of ILC3s produce IL-22, which in turn triggers the anti-microbial peptide production by intestinal epithelium (40–42). Mice with an impairment in the IL-23-ILC3-IL-22 axis succumb to infection by *Citrobacter rodentium*, a gut effacing bacterium (42–44). Furthermore, IL-22 in concert with IL-18 is essential for control of murine norovirus infection (45). Together, these data demonstrate a critical role for ILC3s in maintaining gut homeostasis.

### Innate-like T cells

Innate-like or unconventional T cells express functionally re-arranged T cell receptors (TCRs) of limited diversity. In contrast to conventional T cells whose TCRs strictly recognize peptides in the context of classical polymorphic major histocompatibility molecules (MHCs), the mode of antigen recognition by innate-like T cells is diverse, with TCRs recognizing antigen in the context of canonical MHCs, non-classical non-polymorphic MHC-like molecules, or even independently of MHCs altogether (46). The most well-characterized members of this family of lymphocytes include IELs, iNKT cells, and γδ T cells.

Many epithelial tissues contain resident IEL populations (47). The most studied are the small intestinal IELs, which consist of both TCRαβ- and TCRγδ-expressing subsets (48). The TCRαβ^+^ IELs can be further divided into two major populations based on the surface expression of CD8αβ heterodimer. CD8αβ^−^ IELs, typically expressing the CD8αα homodimer, develop early in life, but its population dwindles as the animal ages and is progressively replaced by CD8αβ^+^ IELs (48). Thus, the CD8αα^+^ subsets are often termed “natural” or “unconventaionl” IELs, to distinguish them from the more conventional CD8αβ^+^ subsets, or the “induced” IELs. In addition to the TCR, CD8αα^+^ IELs also express panoply of activating and inhibitory receptors typically found on innate lymphocytes. These include the Ly49 and other NK receptor family members (49–51). Recently, another subset of IELs, characterized by the expression of both CD4 and CD8αβ co-receptors was identified (52, 53). A series of experiments demonstrate that these CD4^+^CD8αβ^+^ IELs are in fact converted from conventional CD4^+^ T cells by intestinal tissue-specific signals, such as TGFβ (54). So far, the exact functions of IELs remain elusive, although in specific settings, IELs contribute to anti-pathogen responses in the gut (52, 53, 55, 56).

iNKT cells express an invariant TCRα chain paired with a TCRβ chain of limited diversity (46, 57). Distinct from other TCRαβ^+^ T cells, iNKT cells recognize lipid antigens presented in the context of the MHC class I-like molecule, CD1d (58–60). The synthetic glycolipid, alpha-galactosylceramide, has been one of the prototypic stimulators of iNKT cells (61). Since then, a plethora of structurally homologous lipids capable of activating iNKT cells have been identified (62). These range from foreign substances, such as certain bacterial cell wall components (63–65) to endogenous sources, such as intermediates in lipid metabolism (66, 67), although the latter is often only transiently present, rare, and less potent. Nevertheless, sensing of endogenous lipid ligands may be the major mechanism by which iNKT cells detect a breach of tissue integrity. Two studies demonstrate an essential role for iNKT cells in controlling infection by pathogens that lack potent agonist ligands (68, 69), supporting the idea that iNKT cells may primarily survey host cells for altered metabolism as a result of pathogen invasion. Similar to ILCs, iNKT cell subsets analogous to the T_H_1, T_H_2, and T_H_17 conventional CD4 T cells have been described (70). Not unlike these T helper cells, each iNKT cell subset produces its signature cytokines driven by distinct master transcription factors (70).

T cells expressing the TCRγδ are present at barrier sites with a particular enrichment at the skin and intestinal epithelium (71, 72). In mice, rearrangement of the TCRγ locus follows a strict temporal order, resulting in the sequential appearances of distinct γδ T cells bearing monoclonal or oligoclonal TCRs that seed various epithelial tissues during fetal development (71–73). For instance, dendritic epithelial T cells (DETC), characterized by their monoclonal TCR composed of Vγ3 and Vδ1, develop between embryonic days 14 and 16 (73, 74). In contrast, intestinal Vγ7^+^ γδ T cells arise between 2 and 3 weeks after birth (75). It is conceivable that developmental stage-dependent tissue-derived signals permit temporally ordered colonization by distinct γδ T clones. In support of this, two studies demonstrate that *Skint1* and *Btnl* molecules, which are expressed by epithelium during specific stages of development, induce the maturation and potentiate the responses of Vγ5^+^ DETCs and Vγ7^+^ intestinal γδ T cells, respectively (75, 76). The cognate antigens for γδ TCRs are still elusive. Whether MHC molecules are involved in γδ TCR recognition is also unresolved. Similar to innate lymphocytes, γδ T cells rapidly produce cytokines, including interferon gamma (IFNγ) and IL-17, when activated (77). A recent study revealed an unconventional role of skin resident γδ T cells in antagonizing carcinogen-induced melanoma (78). In an IL-4-dependent manner, these γδ T cells promote extrafollicular production of autoreactive IgE, which in turn activate basophils.

### Tissue-resident memory T (T_RM_) cells

The term tissue-resident memory T cells specifically describe populations of conventional T cells that acquire tissue-resident properties. Both CD4 and CD8 T cells can adopt tissue-resident phenotypes (12). Because the CD8^+^ subset has been better characterized, T_RM_ hereafter refers to CD8^+^ T_RM_ cells unless noted otherwise. T_RM_ cells have been commonly regarded as first line of defense in peripheral tissues especially against previously encountered threats (79–81). They are hypothesized to provide timely control of tissue threats before the participation of circulatory memory populations. For instance, a report showed that pre-existing herpes simplex virus (HSV) 2 antigen-specific T_RM_ cells at the vaginal mucosa protect hosts from lethal HSV-2 challenge by restricting viral replication at the site of infection as well as preventing the spread of virus to the peripheral nervous system (81). T_RM_ cells engage in diverse effector functions to mediate host protection. As CD8^+^ T cells can directly lyse infected target cells through the release of granzymes and perforin, several studies reported granzyme B expression in T_RM_ cells as well (19, 23, 82, 83). Notably, T_RM_ cells in the brain can lyse antigen-loaded targets *in situ* (84), suggesting their cytotoxic potential and direct killing as their means of immunosurveillance. By contrast, lung T_RM_ cells protect hosts from influenza virus infection through a process involving IFNγ rather than cytotoxicity (85). More strikingly, recent studies highlighted the innate-like effector property of T_RM_ cells (83, 86, 87). Local activation of T_RM_ cells resulted in their chemokine production, which potently recruited non-antigen specific T cells and initiated an innate immune cascade. Such a bystander response resulted in near-sterilizing immunity against antigentically unrelated pathogens. Thus, in this context, T_RM_ cells can serve as alarm-sounders rather than front line defenders.

## Origin of innate and innate-like tissue-resident lymphocytes

**Figure 1:**
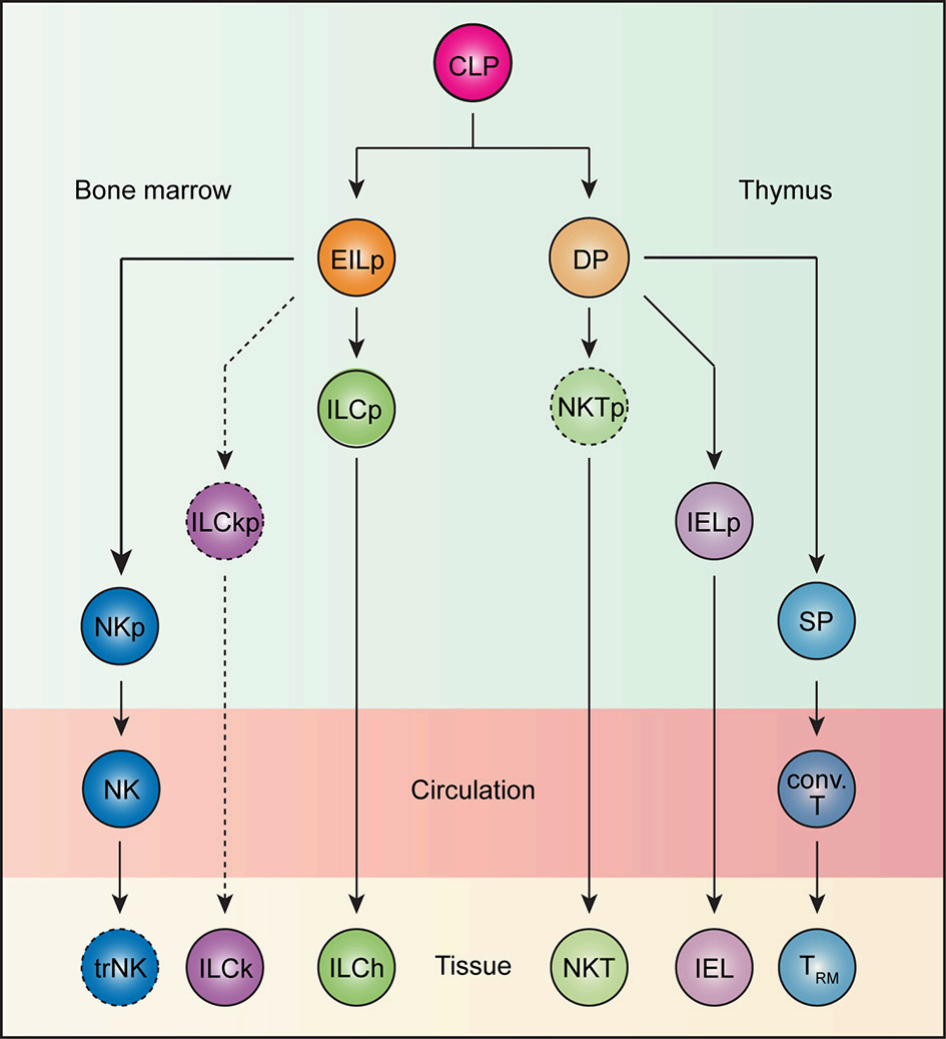
Ontogeny of tissue-resident lymphocytes. All lymphocytes develop from the common lymphoid progenitor (CLP). In the bone marrow, an early innate lymphoid progenitor (EILp) can give rise to natural killer (NK) cells and innate lymphoid cells (ILCs). Whereas, the identity of an NK-restricted progenitor (NKp) remains unknown, a committed innate lymphoid cell progenitor (ILCp), which can give rise to all helper ILCs (ILChs), but not NK cells has been described. Less understood, ILCs with cytotoxic potential, or killer ILCs (ILCk) may arise from a hypothetical killer ILC progenitor (ILCkp) that have lost ILCh and NK potential. While ILCs are inherently tissue-resident, NK cells recirculate. Whether NK cells can acquire tissue-resident features remains unknown. Thus, the term tissue-resident NK (trNK) cells is better kept until such a possibility can be unequivocally ruled out. Beside innate lymphocytes, CLP also gives rise to T lineage-committed progenitors that complete their differentiation in the thymus. The vast majority of TCRαβ-expressing T cells undergo a double positive (DP) stage, during which MHC-based selection takes place. DP thymocytes bearing strongly self-reactive TCRs develop into unconventional intraepithelial lymphocyte (IEL) and natural killer T (NKT) cell lineages through agonist selection, while those with weakly self-reactive TCRs are diverted into single positive (SP) thymocytes, which subsequently give rise to conventional T (conv. T) cells. Whereas, IELs and NKT cells are naturally tissue-resident, conventional T cells recirculate but can become tissue-resident (T_RM_) upon activation.

Adaptive lymphocytes are naturally circulatory and only acquire tissue residency program upon activation. In contrast, innate and innate-like lymphocytes migrate directly to their home tissues after exiting sites of development, bypassing this recirculatory step. We postulate that this difference in trafficking between adaptive and innate/innate-like lymphocytes is imprinted during their development. The developmental pathway of thymocytes to mature T cells is punctuated by several checkpoints, one of which occurs at the double-positive (DP) stage (Figure 1). Here, DP thymocytes test their functionally assembled TCRs for reactivity against self-derived antigens in the context of MHC molecules (88). Strong self-reactivity instructs DP thymocytes to adopt innate-like T cells fates whereas weakly reactive clones are diverted into conventional T cell lineages (88). For instance, thymocytes expressing a transgenic TCR predominantly develop into unconventional IELs when its cognate ligand is expressed in the thymus, but into conventional T cells when otherwise. This process of agonist selection instructs a phenotypic change on DP thymocytes characterized by the downregulation of both CD4 and CD8 co-receptors and the concomitant upregulation of PD-1 (89–92). This population, when adoptively transferred into lymphopenic recipients, exclusively become CD8αα^+^ unconventional IELs, and is thus named IEL progenitor (IELp; Figure 1) (89). Consistently, thymocytes expressing TCRs isolated from natural IELs also adopt the IELp phenotypes (90, 91). In a similar fashion, the endogenous agonist selection ligand, isoglobotrihexosylceramide (iGb3), which strongly stimulates the invariant NKT TCR, drives the lineage commitment of DP thymocytes into iNKT cells (Figure 1) (93). The homotypic interaction between SLAM family receptors is also essential for iNKT development, presumably by complementing TCR-driven selection signals (94, 95). Thus, strong self-reactivity underlies the innate-like T cell fate choice.

Because innate lymphocytes do not express antigen receptors, their self-reactivity is difficult to gage. However, there exist several striking parallels between innate lymphocyte and T cell development. All innate lymphocytes appear to arise from an early innate lymphoid progenitor (EILp; Figure 1). One defining feature of EILp is downregulation of IL-7 receptor (IL-7R), which also occurs in DP thymocytes, presenting a peculiar similarity between the two progenitors among the otherwise IL-7R-dependent intermediates during lymphopoeisis (96, 97). Just as agonist selection signals drive PD1 expression, a PD1-expressing innate lymphoid cell progenitor (ILCp) downstream of EILp has been identified (Figure 1) (35). Like NKT cells, ILCp expresses the transcription factor PLZF and can differentiate into all subsets of helper ILCs (98). The transient upregulation of PD1 on ILCp suggests that all ILCp-derived ILCs engage in a brief but strong stimulation during their development, which parallels the autoreactive TCR-mediated signals that drive IEL commitment. Notably, NK potential is lost in ILCp, although a dedicated NK progenitor remains unidentified (Figure 1) (98). The default circulatory behavior of NK cells aligns them more with the conventional T cells than ILCs. Conceivably, NK cells, like conventional CD8 T cells, may not have experienced a PD1^high^ state during development. In fact, the lack of PD1 expression may help distinguish such NK-dedicated progenitors from their ILC-committed counterparts. The developmental path of cytotoxic ILCs is less understood. In contrast to IL-7R-expressing helper ILCs, which require the transcription factor *Gata3* and *Nfil3* for development, cytotoxic ILCs in the salivary gland are marginally affected upon loss of either transcription factors (29, 31, 99–101). Furthermore, while the vast majority of IL-7R-expressing ILCs develop from the PLZF-expressing ILCP, a substantial fraction of cytotoxic ILCs in the salivary gland do not (102). Additionally, whereas conventional NK cells are critically dependent on *Eomes* and *Nfil3*, cytotoxic ILCs again are not (103–105). These genetic data suggest the existence of yet another innate lymphocyte lineage, which is distinct from both the ILCh and conventional NK cells, and is tentatively named ILCk (Figure 1). ILCks in fact resemble IEL in their constitutive expression of cytotoxic molecules and inherent tissue-resident nature (23). Provocatively, ILCk progenitor may develop from EILp and assume IELp-like phenotypes such as high PD1 but little PLZF expression.

## Acquisition of tissue resident program by circulating lymphocytes

Best exemplified by T_RM_ cells, re-circulating lymphocytes can acquire tissue resident properties upon activation. The exact time point at which the tissue-resident program is launched during the activation history of a T cell is still unknown. Several lines of evidence suggest that tissue tropism of an activated T cells can be imprinted by dendritic cells (DCs) during priming. For instance, T cells activated by DCs isolated from peripheral lymph nodes upregulate E- and P-selectin while those primed by DCs from mesenteric lymph nodes express gut-homing molecules, such as α4β7 integrin and CCR9 (106, 107). Furthermore, the expression of skin- and gut-homing receptors can be enhanced by metabolites specific to these two tissues, such as retinoic acid (108, 109). These data collectively suggest that activated T cells acquire tissue tropism and specific homing capacity during priming. Contrary to this model, recent studies demonstrated that T cell migration is rather promiscuous during the effector phase of the immune response. In fact, T cells primed at any site can access almost every tissue in the organism. For instance, priming of T cells during systemic LCMV infection leads to the migration of antigen-specific T cells to many peripheral tissues (110). More strikingly, intranasal immunization with Sendai virus also results in the migration of antigen-specific T cells to other peripheral tissues (110). Further examination revealed that T cells primed in any secondary lymphoid organs can in fact upregulate homing receptors for non-lymphoid tissues (111). Thus, the entry of a T cell into non-lymphoid tissues can be instated regardless of priming locations. Once inside the tissue, local signals then orchestrate the tissue resident program. Indeed, adoptive transfer of *in vitro* activated CD8 T cells into the dermis is sufficient to induce their differentiation into long-lived CD103^+^CD69^+^ T_RM_ cells, phenotypically indistinguishable from those generated *in vivo* (18). These data suggest that entry into the tissue is a stochastic but pivotal event that marks the initiation of tissue resident program. Recently, fate-mapping experiments using KLRG1-Cre revealed further heterogeneity within the T_RM_ population with contribution from both KLRG1-fate mapped and non-fate mapped precursors (112). This is in contrast to previous studies where KLRG1^+^ CD8 T cells fail to give rise to CD103^+^ T_RM_ when adoptively transferred (18). The discrepancy may be caused by the use of different infection models. Interestingly, although both KLRG1-fate mapped and non-fate mapped precursors lost KLRG1 expression when entering the tissue, the progeny of the two exhibits nuanced but discernable differences in effector functions (112), suggesting that other events before tissue entry can impact the functional capacity of T_RM_.

Often deemed as the counterpart to conventional CD8 T cells, whether NK cells can acquire tissue resident features like T_RM_ differentiation is less understood. In one study, adoptive transfer of hepatic DX5^+^ conventional NK cells into lymphopenic mice did not result in their upregulation of tissue resident markers, such as CD49a in the liver (105). In contrast, when transferred into tumor-bearing lymphopenic recipients, DX5^+^ cells infiltrate the tumor and assume tissue resident phenotypes in a TGFβ-dependent manner (113). These results suggest that re-circulating conventional NK cells possess the tissue resident potential, but its manifestation requires tissue-specific signals. Further studies, such as fate-mapping experiments, are needed to formally test this hypothesis.

## Maintenance of tissue resident lymphocytes

Long-term parabiosis experiments revealed that under steady-state conditions, tissue resident lymphocytes are long-lived and replenish their population predominantly by local expansion (6). Consistently, other studies in mice and rhesus macaques showed that the tissue memory CD8 T cell populations are stable for 300–700 days, with little to no input from the circulatory memory pool (114–116). These observations suggest that while the concerted actions of adhesion molecules and chemokine receptors enforce tissue retention, additional cell-extrinsic signals promote the maintenance of tissue resident lymphocytes.

IL-7 and IL-15, both of which signal through the common gamma chain (γ_c_), have pleiotropic roles during lymphocyte development and maintenance. While mice deficient for γ_c_ (encoded by *Il2rg*) lack B, T, NK, and ILCs, innate lymphocyte progenitors, such as EILp and ILCp were minimally affected (97), suggesting that the depletion of NK and ILCs in *Il2rg*^−/−^ mice most likely stem from defective maintenance of the mature populations. In the absence of IL-7, bone marrow ILC2p, intestinal ILC2 and ILC3, but not ILC1 are drastically reduced (97, 117–119). In contrast, IL-15 deficiency predominantly impairs ILC1 in the liver, salivary glands, and the small intestine lamina propria (29, 119, 120), although intestinal NKp46^+^ ILC3 are dually dependent on IL-7 and IL-15 (119, 120). While the NK-restricted progenitor remains elusive, a Lin^−^CD127^+^CD122^+^ population has been identified to contain NK cell precursors and develop normally in the absence of *Il2rg* (121). The profound ablation of mature CD127^−^ NK cells in these animals are attributed to the lack of IL-15 signaling as IL-15, but not other γ_c_ cytokines, deficiency can solely recapitulate this defect (121–123). In the thymus, a minute population of CD127^+^NK1.1^+^ innate lymphocytes, currently called thymic NK cells, require IL-7 for development (124).

The critical roles of homeostatic cytokines IL-7 and IL-15 for the maintenance of re-circulating naïve and memory T cells, respectively have been long appreciated. The dependency on IL-15 for T_RM_ varies by their locations. T_RM_ in the non-lymphoid tissues, such as the skin, are critically dependent on IL-15 (18) whereas those in the secondary lymphoid organs are not (125). Like T_RM_, CD8αα^+^ intestinal IELs are also maintained by IL-15 and enterocyte-expressed IL-15 in an otherwise IL-15-deficient animal is sufficient to restore unconventional IELs (126), suggesting that IL-15 critically sustains mature IELs rather than their precursors. In support of this, PD1^+^ IEL progenitors develop independent of IL-15 in the thymus (127). While T_RM_ are induced in an antigen-dependent manner, they can be maintained in the absence of cognate antigen in the skin, reproductive tract, and salivary glands (18, 19, 21). In other tissues, persisting antigens contribute to T_RM_ differentiation (19, 26, 82, 84, 128). Thus, the requirement for antigen during T_RM_ maintenance may be tissue-specific. Lastly, given the similar requirement for IL-7 and IL-15 during their homeostasis, resident lymphocytes may occupy overlapping tissue niche. Pinpointing the source of these cytokines in the tissue may help elucidate the redundant and non-redundant roles of each resident lymphocyte population in maintaining tissue integrity.

## Tissue-resident cytotoxic lymphocyte responses in cancer

**Figure 2:**
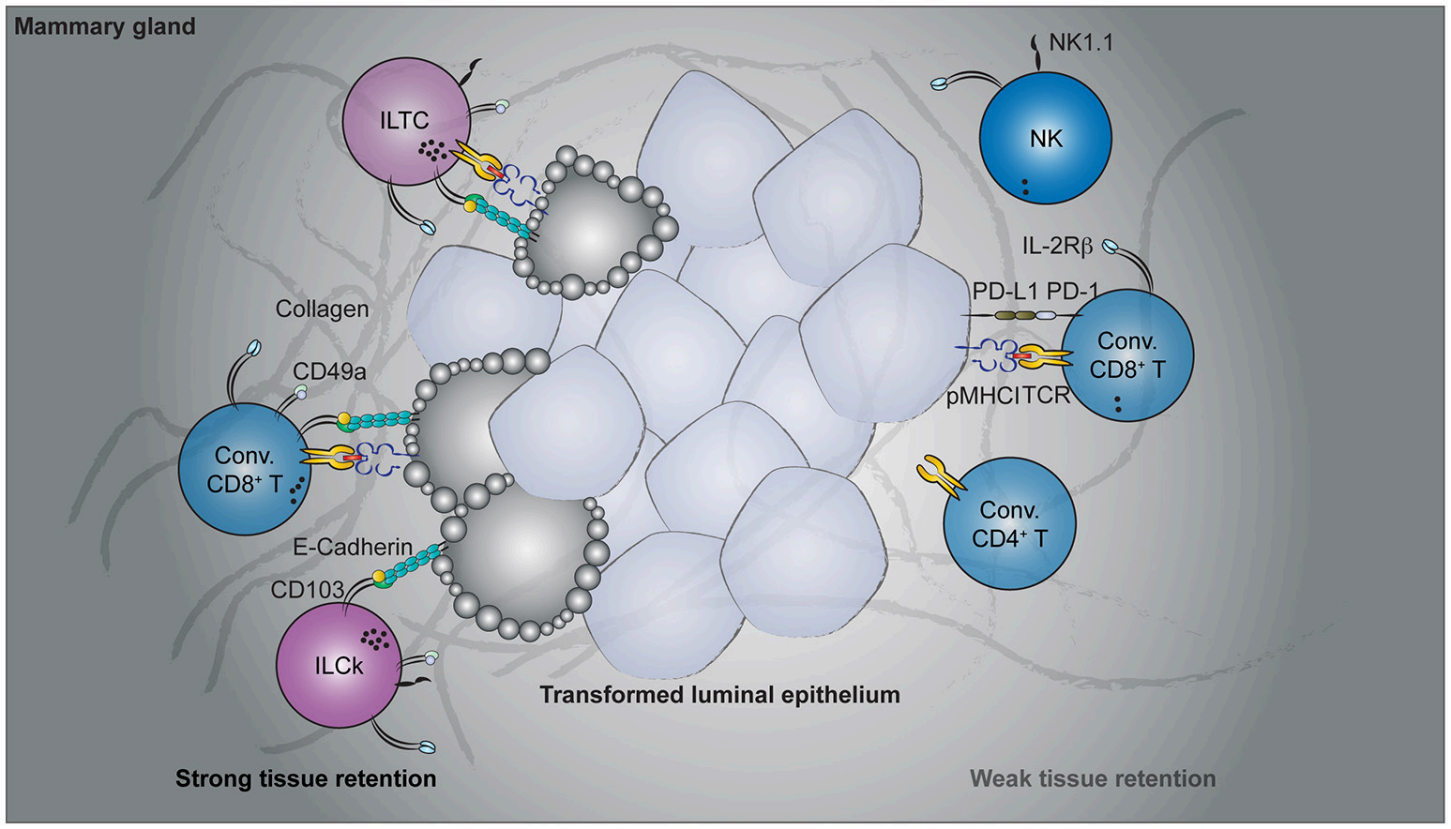
Cancer immunosurveillance by tissue-resident lymphocytes. Spontaneous oncogene-driven breast tumors are infiltrated by group 1 innate lymphocytes, conventional, and unconventional T cells. Parabiosis experiments revealed the tissue-resident nature of CD49a- and CD103-co-expressing lymphocytes, including the innate-like T cells (ILTCs), killer innate lymphoid cells (ILCks), and some conventional (Conv.) CD8^+^ T cells. In contrast, natural killer (NK) cells, PD1-expressing conventional CD8^+^ T cells recirculate through blood. Functionally, CD49a^+^CD103^+^ tissue-resident lymphocytes abundantly express lytic granules and can potently lyse transformed target cells. Despite their cytotoxicity, therapies targeting these tissue-resident populations are lacking while rapid advancement has been made to target conventional NK and T cells.

The vertebrate immune system has evolved to exquisitely distinguish self from non-self, thereby achieving effective anti-pathogen responses while curbing autoreactivity. Cancer presents a unique challenge to this fine-tuned system as transformed cells are pathogenic agents derived from the host itself. Yet prevailing evidence has demonstrated that the immune system exerts constant pressure on tumors (129). These observations underlie the preponderant concept of cancer immunosurveillance (130–132). Mechanistically, increased somatic mutation as a result of genomic instability in transformed cells may generate neo-epitopes that can be recognized by conventional adaptive lymphocytes (133, 134). Although these T cells often exhibit “exhausted” phenotypes, their effector functions may be restored by checkpoint blockade therapies (134–136) (Figure 2). Targeting this mode of immunosurveillance certainly has been fruitful. However, not all cancer types sustain high mutation burden (137, 138). In such cases, CD8 T cell responses elicited by unmutated self-antigen often fail to restrict tumor growth (139, 140). These findings thus highlight the need to explore other immunosurveillance mechanisms for effective cancer immunotherapies.

Just as pre-existing T_RM_ populations are essential for restraining previously encountered pathogens, prophylactically induced T_RM_ cells by cancer vaccines provide superior control of tumor growth over re-circulating memory T cells (141, 142). In fact, the presence of circulating tumor antigen-specific CD8 T cells alone is not sufficient to control tumor growth (141, 143), highlighting the potential therapeutic benefit of targeting tissue-resident lymphocytes. Strategies to enhance the differentiation and maintenance of these vaccine-induced T_RM_ cells may decrease the relapse rate as well as restrict metastasis. However, prophylactic vaccination with tumor-associated antigen may not always be feasible in clinical settings, as it requires knowing the antigen ahead of time when patients who seek medical attention often have developed tumors already. Notwithstanding, tumorigenesis does naturally elicit tissue-resident lymphocyte responses (23, 144–148). Importantly, a substantial fraction of participating lymphocyte populations appear to have cytotoxic potential (23, 145, 148). These include conventional T cells of the CD8 lineage as well as more recently identified unconventional T cells and group 1 innate lymphocytes (Figure 2). Below, we summarize the latest findings on their characterization and potential cancer immunosurveillance functions.

### Conventional and unconventional αβT cells

In many murine tumor models, αβ T cells can make up a substantial fraction of infiltrating lymphocytes. Among them, populations expressing tissue-resident markers are abundantly found (23, 144, 145, 148–152). These include T cells of both the conventional and unconventional lineages.

Our understanding of tissue-resident T cell responses in the context of cancer has only begun to advance in recent years. Much of the foundation is in fact built upon extrapolating observations from T_RM_ cells in infectious settings. While these studies provide an invaluable conceptual framework to start with, cancer and acute infection differ fundamentally. Tumorigenesis is a continuous process without a defined time course. In contrast to acute infections where the pathogen load peaks and wanes within a week's time, tumor-associated antigen is continuously present and, in most oncogene-driven cancer models, persist until the endpoint of disease. Thus, there is no well-defined memory phase in the context of cancer and the term “tissue-resident memory T cells” seems to be a misnomer. In a sense, tumorigenesis is more analogous to chronic than acute infections. Indeed, the induction and accumulation of dysfunctional cytotoxic T lymphocytes (CTLs) by persistent antigen stimulation is a shared feature in both settings (153). To what extent the PD1^hi^ CTLs are tissue-resident remains to be determined. Beside the PD1^hi^ population, which appears to dominate in multiple cancer types, tumor-infiltrating CD8^+^ T cells that express tissue-resident markers have also been reported in several mouse cancer models (Figure 2). In a B16-F10 mouse melanoma transplantable tumor model, a fraction of antigen-specific tumor-infiltrating CD8 T cells acquired CD69 and CD103 expression 3 weeks after tumor engraftment (149). Furthermore, administration of blocking antibodies against CD103 resulted in a slight but significant acceleration in tumor growth (149), implying a CD103-dependent cancer immunosurveillance mechanism by these putative tissue-resident tumor-infiltrating lymphocytes (TILs). Using a similar transplantable melanoma model, another study demonstrated a CD8 T cell-intrinsic requirement for the transcription factor *Runx3* in the development of tumor-resident CTL responses (144). CD8 T cells with reduced levels of *Runx3* expression failed to constrain tumor growth (144), further implicating a tumor surveillance role for tissue-resident CTLs. In a spontaneous oncogene-driven breast tumor model, a proportion of intratumoral CD8^+^ T cells co-express CD49a and CD103 (23). Unlike in the transplantable tumor models, some CD49a^+^CD103^+^ T cells co-express natural killer (NK) receptors, such as NK1.1 and have innate-like features (Figure 2). These NK1.1^+^CD49a^+^CD103^+^CD8^+^ T cells are distinct from iNKT cells as they developed in the absence of CD1d, and thus represent a novel tissue-resident T cell population with no currently known counterpart in the T_RM_ field (23). For this, NK1.1^+^CD49a^+^CD103^+^CD8^+^ T cells are termed innate-like T cells (ILTCs) to distinguish them from their NK1.1^−^ counterparts. Parabiosis experiments confirmed the tissue-resident property of both ILTCs and NK1.1^−^ tumor-infiltrating T cells, with the former being significantly less circulatory (23). Further studies demonstrated that these ILTCs produce little to no IFNγ, but abundantly express the cytotoxic molecule granzyme B (23). Indeed, ILTCs exhibit potent cytotoxicity toward transformed target cells *in vitro*, suggesting their potential role in anti-tumor responses (23). Thus, using infection-induced T_RM_ cells as a template, these seminal works demonstrated the presence of tissue-resident cytotoxic T cells in mouse tumor models and implicated their immunosurveillance functions.

In human patients, CD103-expressing tumor infiltrating CD8^+^ T cells are abundantly present in multiple types of epithelium-derived cancers (145–147). In many cases, the accumulation of intratumoral CD103^+^CD8^+^ T cells is associated with favorable prognosis (145–147, 154, 155). Although the exact mechanisms by which these TILs contribute to restraining cancer progression remains elusive, emerging evidence unveil their similarity to T_RM_ cells and suggest cytotoxicity as their mechanism of immunosurveillance. Whether CD103^+^ TILs are indeed tissue-resident cannot be easily established in humans. Nonetheless, whole genome transcriptome analysis reveals that these TILs share a gene expression program typically associated with pathogen-induced T_RM_ cells and tumor-elicited CD49a^+^CD103^+^ TILs in mouse models (23, 142, 148, 156). For instance, CD103^+^ TILs from non-small cell lung carcinoma co-express CD49a and CD69, but little to no S1PR1 and the lymphoid tissue homing receptor CCR7 (145, 148). In addition to potentially increased tissue retention, CD103^+^ TILs appear to be in a distinct activation state compared to their CD103^−^ counterparts. Not only do more CD103^+^ TILs exit quiescence, as measured by Ki67 expression (148), they also express higher levels of granzymes (148) and possess increased degranulation potential relative to CD103^−^ TILs in response to stimulation (145). When incubated with autologous tumor cells, CD103^+^ TILs potently induced cytolysis of target cells (145). Whether this CD103^+^ population also contains innate-like T cells, such as the ILTCs found in mice, remains an outstanding question although NK receptor-expressing CD8^+^ T cells in human cancer patients have been documented (157–159). Nevertheless, these data demonstrate that the tissue-resident cytotoxic T cell response is a conserved cancer immunosurveillance mechanism between mouse and human and represents a promising target for tumor immunotherapy.

### Group 1 innate lymphocytes

The protective role of group 1 innate lymphocytes against tumors has been repeatedly demonstrated in chemically-induced sarcoma and transplantable tumor models (160–163). However, most of these seminal works were done before the distinction between NK cells and ILCs was recognized. Most studies in this genre made use of depleting antibodies against NK1.1 or genetic systems in which diphtheria toxin is specifically expressed in NKp46^+^ cells. These approaches effectively eliminated NK cells, but also depleted ILC1s and ILCks as they too express NK1.1 and NKp46. Thus, one cannot conclude which of the affected population contributes to the reported phenotype (164). Having recognized this ambiguity, some studies further subset the NK1.1^+^NKp46^+^ innate lymphocyte populations with a set of markers conventionally used to distinguish between NK cells and ILC1s/ILCks (113, 165). Adoptive transfer of each subset into tumor-bearing lymphopenic hosts then allowed them to identify the population responsible for the protective phenotypes. In these studies, most anti-tumor activity appears to reside within the conventional NK cell compartment (75, 113). Non-NK tissue-resident innate lymphocytes, on the other hand, were shown to dampen anti-tumor immune responses (113). This is in contrast to their roles in oncogene-driven spontaneous tumor models (23, 166). For example, in a breast tumor model, early control of tumor progression is critically dependent on innate lymphocytes, as IL-15 deficient animals, which lack group 1 innate lymphocytes showed accelerated tumor growth (23). However, conventional NK cells were dispensable for this innate lymphocyte-dependent anti-tumor responses because *Nfil3*-deficient mice, which have profoundly diminished NK cell compartment, did not exhibit accelerated tumor growth (23). These data collectively imply that non-NK group 1 innate lymphocytes, most likely ILCks, assume a dominant role in early anti-tumor responses (Figure 2). Despite these tumor model-specific discrepancies, the immunosurveillance potential of tumor-infiltrating group 1 innate lymphocytes has garnered much therapeutic interest in recent years.

Many types of human solid tumors are also infiltrated by group 1 innate lymphocytes. Although collectively called NK cells, they in fact consist of two populations distinguished by the makers CD56 and CD16 (167–170). The CD56^bright^CD16^−^ subset outnumbers their CD56^dim^CD16^+^ counterpart in tissues, both at steady state and during inflammation. In contrast, the CD56^dim^CD16^+^ population is far more abundant in the blood. Not surprisingly, the CD56^bright^CD16^−^ innate lymphocytes express several tissue-resident markers as well as a defining gene expression program for tissue residency (169, 170). Under the current paradigm, both populations belong to the NK lineages and are related in a linear developmental pathway, namely, CD56^bright^CD16^−^ cells give rise to CD56^dim^CD16^+^ in a process of differentiation (171, 172). However, it is also possible that the two populations are in fact of disparate lineages, a distinction not unlike the one seen between mouse NK cells and ILC1s/ILCks. While this debate awaits, if possible, a resolution, some clinical evidence suggest a potential anti-tumor role for type 1 innate lymphocytes. For example, in clear cell renal carcinoma, enrichment of type 1 innate lymphocyte-associated transcripts in the tumor mass correlates with favorable prognosis (173). Similarly, for gastrointestinal stroma tumors, the number of CD56-expressing infiltrating lymphocytes is associated with better overall survival (174). For patients with non-small cell lung carcinoma however, the presence of CD56-expressing lymphocytes does not correlate with clinical outcomes, presumably because their cytokine production and cytotoxicity are inhibited by the tumor microenvironment (175). Overcoming immunosuppression strategies deployed by tumor cells may re-invigorate these innate lymphocytes (176–178). A recent study devised an antibody that stabilizes the expression of a stress-induced ligand for the NK activating receptor, NKG2D on the tumor cell surface (179). Administration of this therapeutic agent enhances innate lymphocyte-dependent anti-tumor responses (179). Collectively, tumor-resident cytotoxic innate lymphocytes present a promising target for therapeutic intervention in addition to conventional CD8 T cells, for which a plethora of checkpoint blockade modalities are already in place.

## Concluding remarks

Originally defined in the T cell field, the tissue residency program has now been found to be used by nearly all known lymphocyte lineages across the hematopoietic tree. Intriguingly, the vast majority of innate and innate-like lymphocytes (with the exception of NK cells) are inherently tissue-resident whereas the more recently evolved adaptive lymphocytes are not, suggesting an ancient origin of the tissue residency program. Since strong self-reactivity during lymphocyte development appears to be a key selection factor for gaining tissue-homing capacity, it is reasonable to assume that the most primordial function of tissue-resident lymphocytes is in fact to detect stress in host cells rather than to sense pathogen or its derivatives. Further extrapolation of this idea would provocatively suggest that the MHC-based selection mechanisms originally served to generate self-reactive T cells. Positive selection, templated on the extant agonist selection mechanisms, evolved later in vertebrate evolution.

## Author contributions

CC and ML conceived the ideas. CC wrote the manuscript, and ML edited it.

### Conflict of interest statement

The authors declare that the research was conducted in the absence of any commercial or financial relationships that could be construed as a potential conflict of interest.
